# Impact of liver metastases status on survival outcomes of first-line immunotherapy in extensive stage small cell lung cancer: a systematic review and meta-analysis

**DOI:** 10.18632/aging.205035

**Published:** 2023-09-18

**Authors:** Yan Lin, Wei Jiang, Cui-Yun Su, Xin-Bin Pan

**Affiliations:** 1 Department of Gastroenterology, Jiangbin Hospital of Guangxi Zhuang Autonomous Region, Nanning 530000, Guangxi, P.R. China; 2 Department of Respiratory Oncology, Guangxi Medical University Cancer Hospital, Nanning 530021, Guangxi, P.R. China; 3 Department of Radiation Oncology, Guangxi Medical University Cancer Hospital, Nanning 530021, Guangxi, P.R. China

**Keywords:** small cell lung cancer, SCLC, extensive stage, immunotherapy, liver metastases

## Abstract

Purpose: This study aims to assess the impact of liver metastases status on survival outcomes of first-line immunotherapy in extensive stage small cell lung cancer (ES-SCLC) patients.

Materials and methods: Comprehensive searches were conducted in the Cochrane Library databases, Embase, PubMed, and abstracts from WCLC, ESMO, and ASCO from inception to December 2022. Randomized controlled trials reporting progression-free survival (PFS) and/or overall survival (OS) of first-line immunotherapy in ES-SCLC patients were included.

Results: Six trials involving 3501 patients were analyzed, comprising 1350 patients with liver metastases and 2151 without. The quality of the included trials was consistently high. Pooled results revealed that immunotherapy plus chemotherapy did not significantly improve PFS (hazard ratio [HR] = 0.82, 95% confidence interval [CI]: 0.68-1.00, P = 0.05) and OS (HR = 0.89, 95% CI: 0.79-1.00, P = 0.05) in ES-SCLC patients with liver metastases compared to chemotherapy alone. However, immunotherapy plus chemotherapy improved PFS (HR = 0.66, 95% CI: 0.57-0.77, P < 0.01) and OS (HR = 0.74, 95% CI: 0.67-0.82, P < 0.01) in ES-SCLC patients without liver metastases compared to chemotherapy alone.

Conclusions: First-line immunotherapy plus chemotherapy significantly improved PFS and OS in ES-SCLC patients without liver metastases compared to chemotherapy alone. However, patients with liver metastases did not experience comparable benefits.

## INTRODUCTION

Small cell lung cancer (SCLC) is a highly aggressive neuroendocrine malignancy [1]. The rapid growth rate of SCLC leads to about 75% of cases being diagnosed at an advanced stage with distant metastases [2, 3]. Among these cases, approximately 25% of extensive stage SCLC (ES-SCLC) patients present with liver metastases [4, 5].

Immunotherapy combined with chemotherapy has shown potential for improved survival outcomes as a first-line treatment for ES-SCLC patients [6–12]. However, variations in the efficacy of immunotherapy based on liver metastases status have raised important questions. This systematic review and meta-analysis aims to assess the survival outcomes of first-line immunotherapy in ES-SCLC patients, considering liver metastases status.

## MATERIALS AND METHODS

### Trial search

In accordance with the Preferred Reporting Items for Systematic Reviews and Meta-Analyses guidelines [13, 14], two authors (Yan Lin and Wei Jiang) independently conducted thorough searches across various databases including the Cochrane Library, Embase, PubMed, as well as abstracts from World Conference on Lung Cancer, European Society of Medical Oncology, and American Society of Clinical Oncology, spanning from inception to December 2022. Key search terms included small cell lung cancer, SCLC, extensive stage, stage IV, randomized controlled trial, and RCT. Furthermore, references from significant clinical trials were also screened.

### Trial selection

Trials meeting criteria including randomized controlled design, histologically/cytologically confirmed SCLC, extensive stage, reporting of immunotherapy and chemotherapy survival outcomes, and provision of hazard ratios (HRs) with 95% confidence intervals (CIs) for progression-free survival (PFS) and/or overall survival (OS) in patients with liver metastases were included.

### Trial quality assessment

The methodological quality of trials was independently assessed by two authors (Yan Lin and Wei Jiang) using the Cochrane Risk of Bias tool [15]. Any discrepancies were resolved through discussion or consultation with a third researcher (Xin-Bin Pan).

### Data extraction

Data from included trials were extracted by two authors (Cui-Yun Su and Xin-Bin Pan), adhering to the Preferred Reporting Items for Systematic Reviews and Meta-Analysis guidelines.

### Statistical analysis

Estimated HRs of PFS and OS along with their respective 95% CI limits were calculated and illustrated through forest plots. Sensitivity analyses were carried out to gauge the impact of excluding specific trials. Begg’s and Egger’s tests were used to assess publication bias. Random effect models were employed in the presence of statistical heterogeneity (I^2^ ≥ 50%, P < 0.10), while fixed effect models were utilized in its absence.

Statistical analyses were performed using R software version 4.3.0 and SPSS Statistics Version 26.0 (IBM Co., Armonk, NY, USA). A significance level of P < 0.05 was adopted.

## RESULTS

### Characteristics of included trials

**Figure 1:**
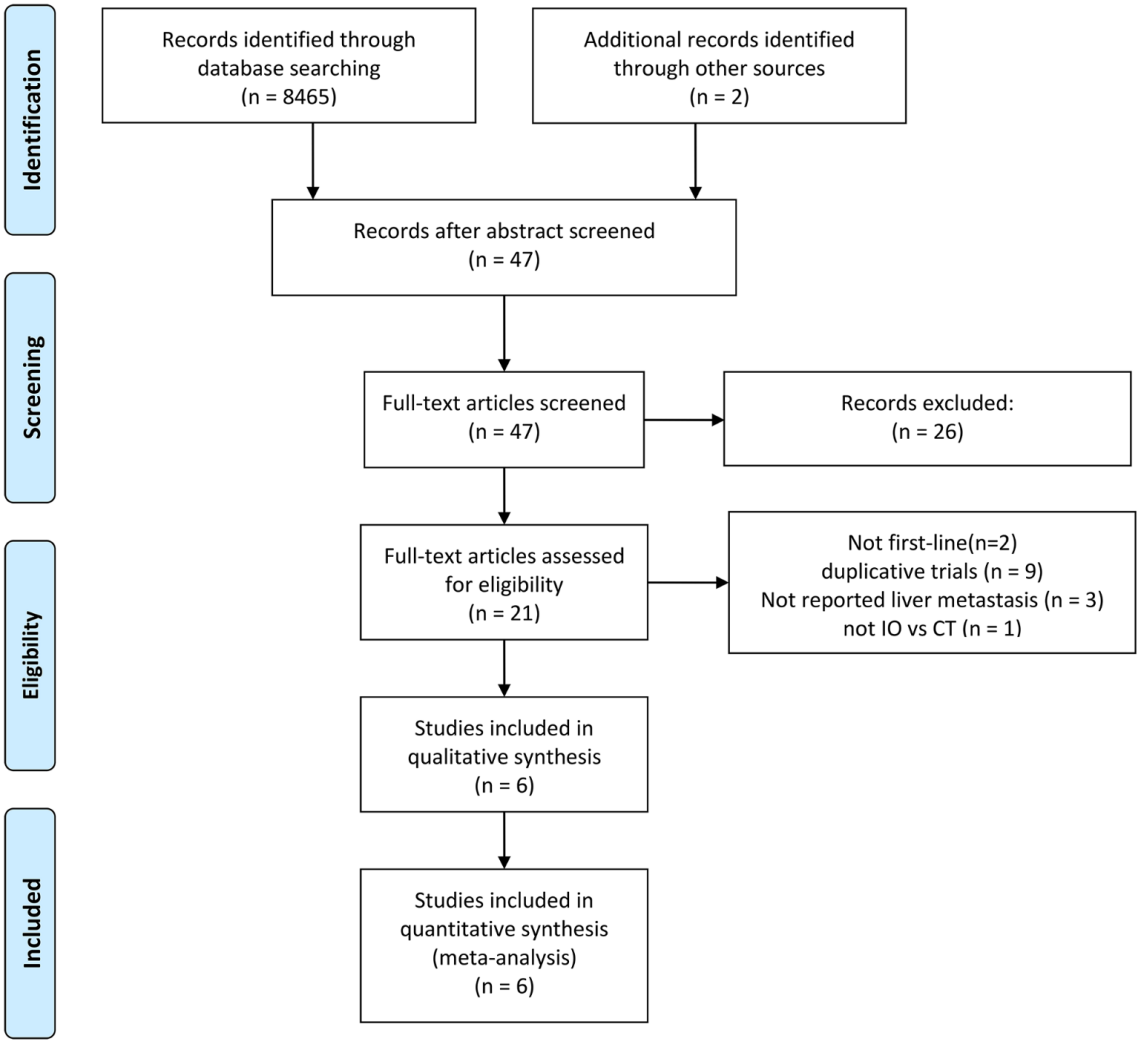
**Flowchart illustrating trial selection.** IO: immunotherapy. CT: chemotherapy.

**Figure 2:**
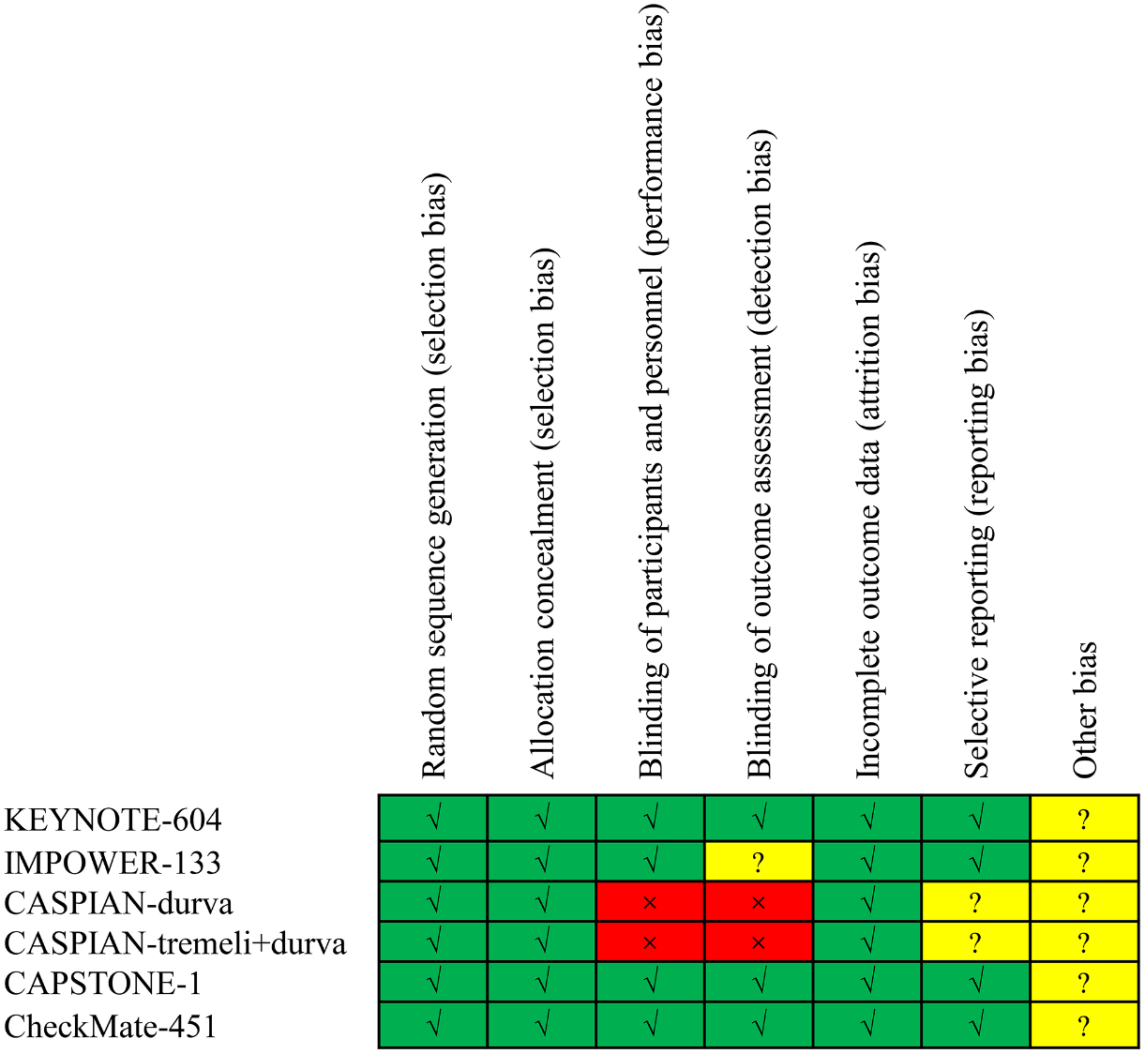
Risk of bias assessment for the included trials.

Following the evaluation of 8467 studies, 6 phase III randomized clinical trials were incorporated into the analysis [7, 9–12, 16]. The trial selection process is visually represented in Figure 1. These trials encompassed 3501 ES-SCLC patients, with 1350 having liver metastases and 2151 without. A detailed account of trial characteristics is presented in Table 1, while the methodological quality is delineated in Figure 2. The quality of the included trials was consistently high.

**Table 1:** Baseline characteristics of the included studies.

**Trials**	**Year**	**Area**	**Phase**	**Treatments**	**Liver metastases**	
					**Yes**	**No**
KEYNOTE-604	2020	worldwide	3	Pembrolizumab+EP/EC q3w×4	95	133
				EP/EC q3w×4	92	133
IMpower-133	2018	worldwide	3	Atezolizumab+EC q3w×4	77	124
				EC q3w×4	72	130
CASPIAN-durva	2019	worldwide	3	Durvalumab+EP/EC q3w×6	108	160
				EP/EC q3w×6	104	165
CASPIAN-tremeli+durva	2020	worldwide	3	Durvalumab+Tremelimumab+EP/EC q3w×6	117	151
				EP/EC q3w×6	104	165
CAPSTONE-1	2022	China	3	Adebrelimab+EC q3w×4-6	73	157
				EC q3w×4-6	74	158
ChechMate-451-nivo	2021	worldwide	3	Nivolumab+EP/EC q3w×3-4	106	174
				EP/EC q3w×3-4	109	166
ChechMate-451-ipili+nivo	2021	worldwide	3	Ipilimumab+nivolumab+EP/EC q3w×3-4	110	169
				EP/EC q3w×3-4	109	166

EP, etoposide+cisplatin; EC, etoposide+carboplatin; Durva, durvalumab; Tremeli, tremelimumab; Nivo, nivolumab; Ipili, ipilimumab.

### Pooled HRs for PFS

**Figure 3:**
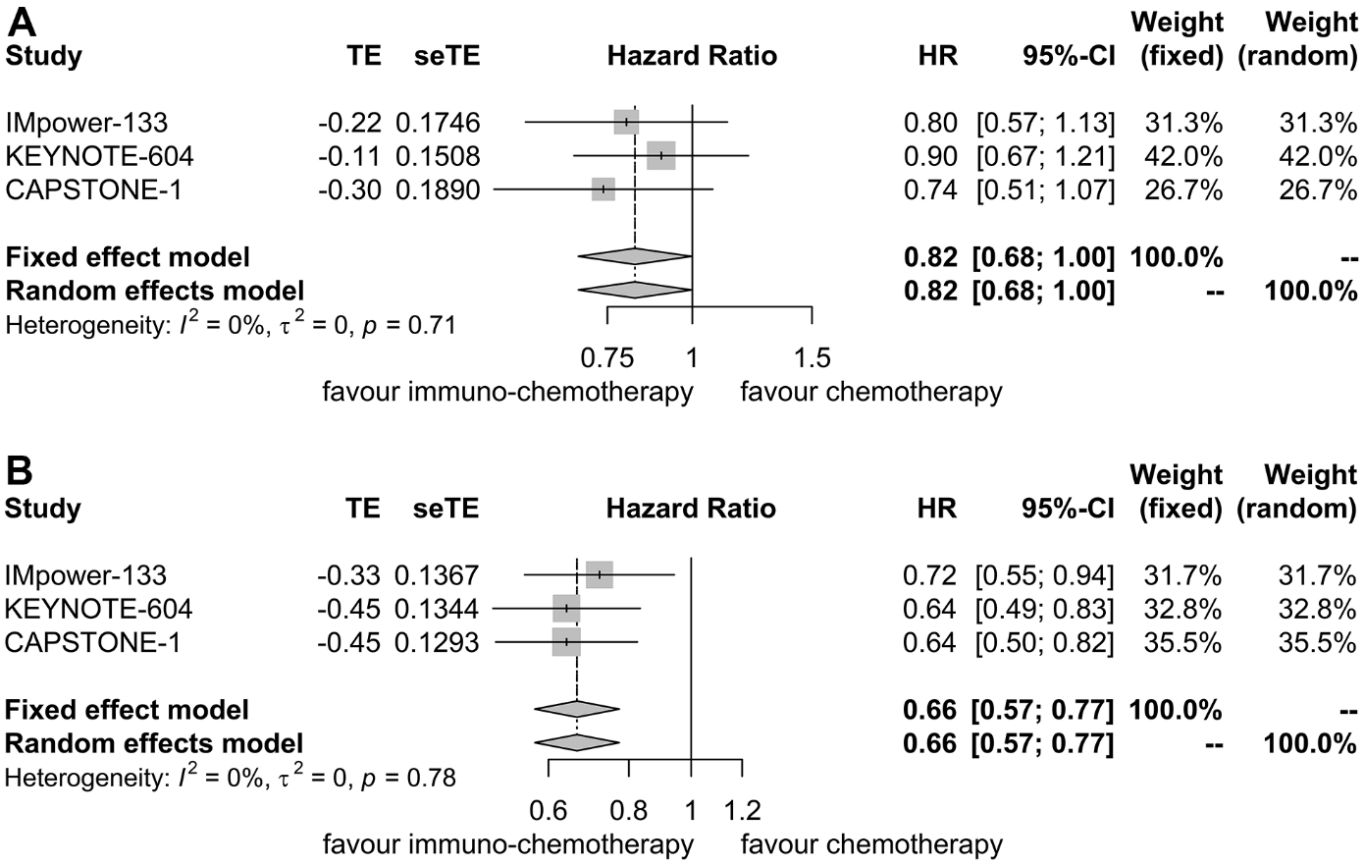
**Forest plot displaying hazard ratios for comparing progression-free survival between immuno-chemotherapy and chemotherapy.** (**A**) Patients with liver metastases. (**B**) Patients without liver metastases.

For patients with liver metastases, PFS HR was reported in three trials [7, 11, 12]. No heterogeneity was detected (I^2^ = 0.00%, P = 0.71), warranting the application of a fixed effect model. Immunotherapy plus chemotherapy did not yield a significant improvement in PFS when compared to chemotherapy alone (HR = 0.82, 95% CI: 0.68-1.00, P = 0.05; Figure 3A).

Similarly, for patients without liver metastases, PFS HR was reported in three trials as well [7, 11, 12]. No heterogeneity was observed (I^2^ = 0.00%, P = 0.78), prompting the use of a fixed effect model. Immunotherapy plus chemotherapy demonstrated improved PFS compared to chemotherapy alone (HR = 0.66, 95% CI: 0.57-0.77, P < 0.01; Figure 3B).

**Figure 4:**
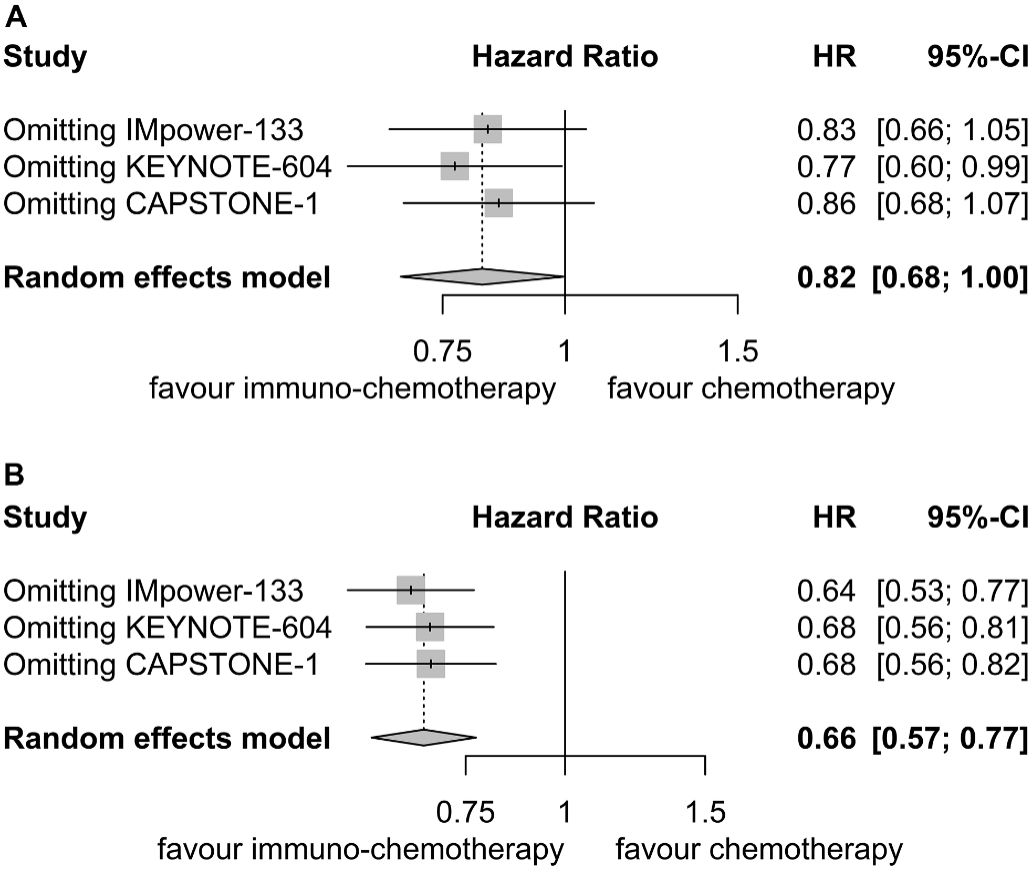
**Sensitivity analysis for progression-free survival.** (**A**) Patients with liver metastases. (**B**) Patients without liver metastases.

**Figure 5:**
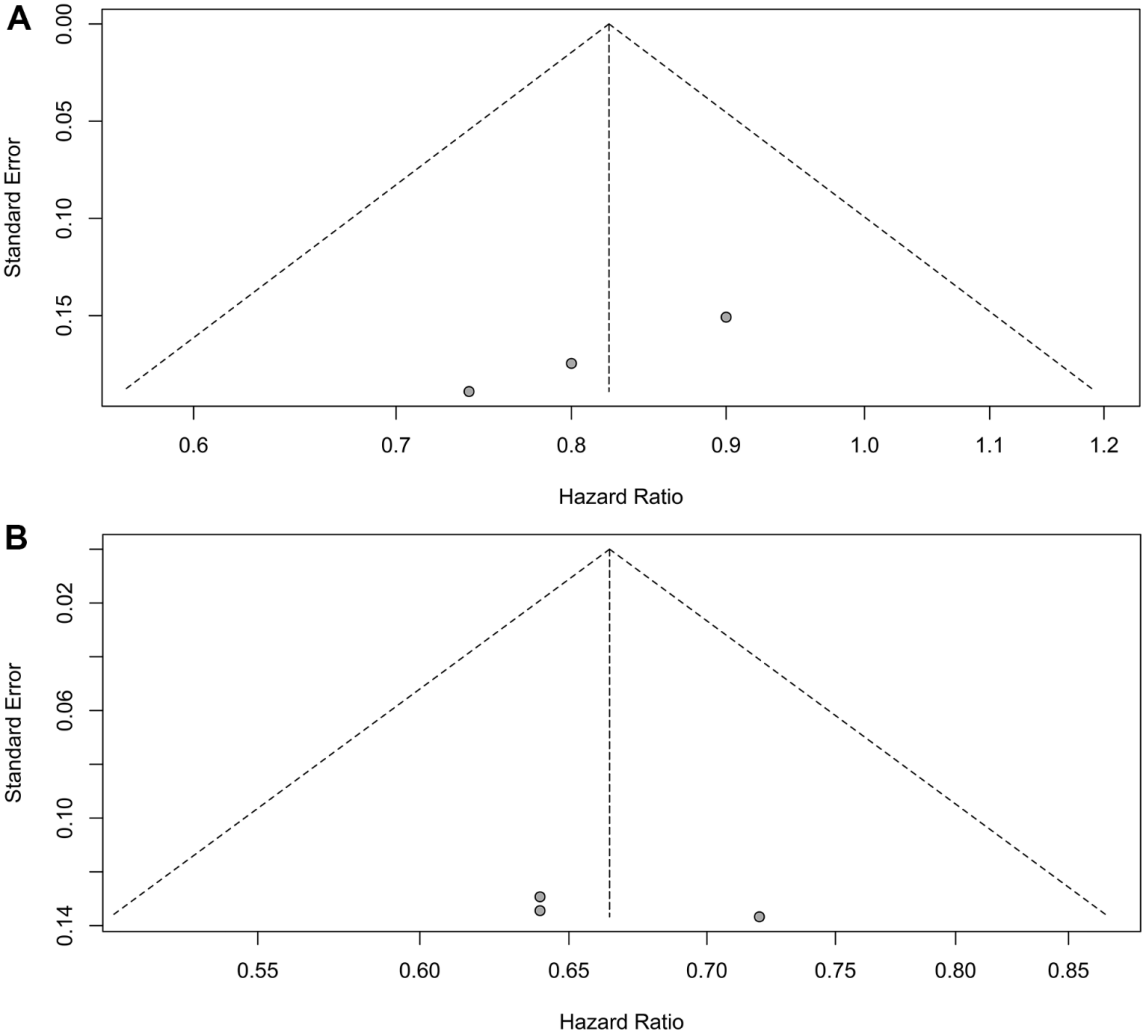
**Assessment of publication bias for progression-free survival.** (**A**) Patients with liver metastases. (**B**) Patients without liver metastases.

Sensitivity analyses confirmed the robustness of these findings. Figure 4A displays the sensitivity analyses of PFS for patients with liver metastases, while Figure 4B exhibits the sensitivity analyses of PFS for patients without liver metastases. No discernible publication bias was detected, as evidenced by the results of Egger’s (P = 0.92) and Begg’s (P = 0.98) tests across the three randomized clinical trials involving patients with liver metastases (Figure 5A). Similarly, among the three randomized clinical trials involving patients without liver metastases, Egger’s (P = 0.90) and Begg’s (P = 0.94) tests yielded no conspicuous indications of publication bias (Figure 5B).

### Pooled HRs for OS

**Figure 6:**
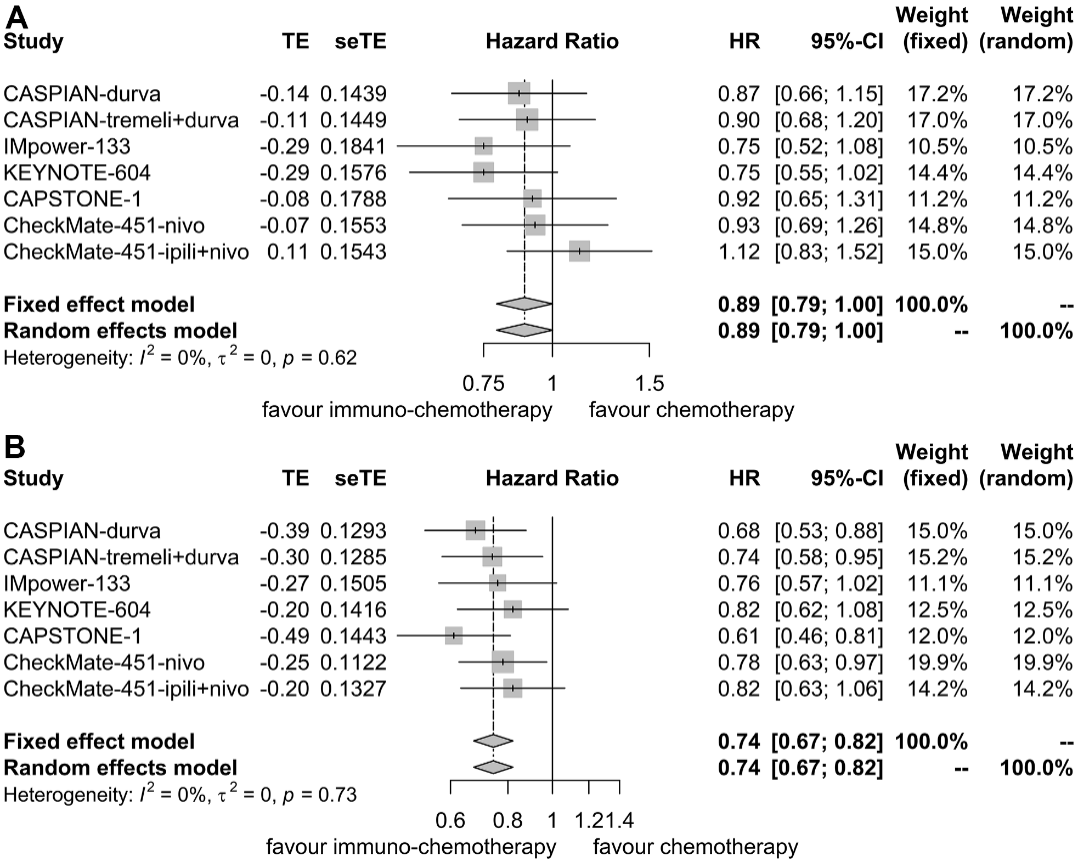
**Forest plot illustrating hazard ratios for overall survival between immuno-chemotherapy and chemotherapy.** (**A**) Patients with liver metastases. (**B**) Patients without liver metastases.

OS HR was reported in 6 trials for patients with liver metastases [7, 9–12, 16]. No heterogeneity was observed (I^2^ = 0.00%, P = 0.62), leading to the application of a fixed effect model. Immunotherapy plus chemotherapy did not substantially enhance OS when compared to chemotherapy alone (HR = 0.89, 95% CI: 0.79-1.00, P = 0.05; Figure 6A).

Similarly, for patients without liver metastases, OS HR was reported in 6 trials as well [7, 9–12, 16]. No heterogeneity was detected (I^2^ = 0.00%, P = 0.73), resulting in the utilization of a fixed effect model. Immunotherapy plus chemotherapy demonstrated improved OS compared to chemotherapy alone (HR = 0.74, 95% CI: 0.67-0.82, P < 0.01; Figure 6B).

**Figure 7:**
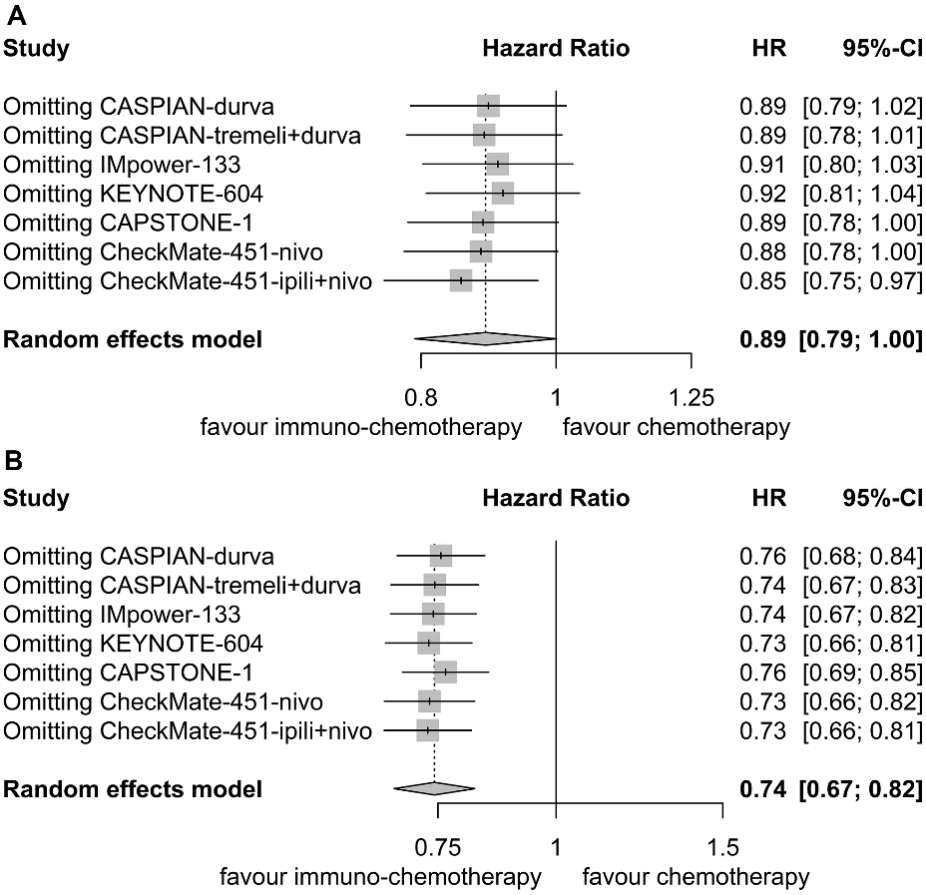
**Sensitivity analysis for overall survival.** (**A**) Patients with liver metastases. (**B**) Patients without liver metastases.

**Figure 8:**
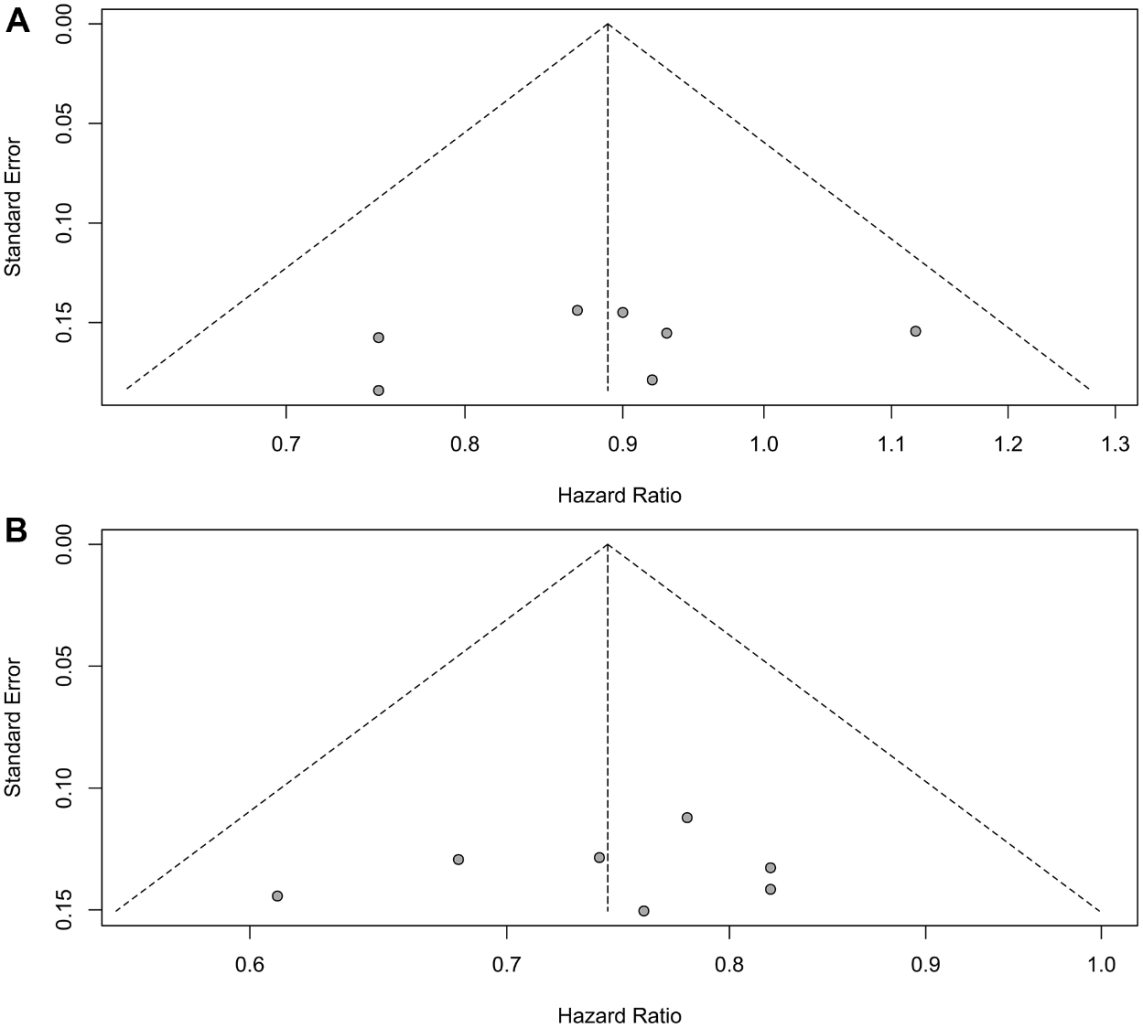
**Assessment of publication bias for overall survival.** (**A**) Patients with liver metastases. (**B**) Patients without liver metastases.

Sensitivity analyses confirmed the robustness of these findings. Figure 7A illustrates the sensitivity analyses of OS for patients with liver metastases, while Figure 7B presents the sensitivity analyses of OS for patients without liver metastases. There was no noticeable indication of publication bias, as indicated by the results of Egger’s (P = 0.53) and Begg’s (P = 0.65) tests across the six randomized clinical trials involving patients with liver metastases (Figure 8A). Similarly, among the six randomized clinical trials involving patients without liver metastases, Egger’s (P = 0.61) and Begg’s (P = 0.65) tests revealed no apparent presence of publication bias (Figure 8B).

## DISCUSSION

This systematic review and meta-analysis concluded that first-line immunotherapy plus chemotherapy significantly enhanced PFS and OS in ES-SCLC patients without liver metastases compared to chemotherapy alone. However, for patients with liver metastases, the benefits were not as pronounced. These findings emphasized the need for further exploration into the immunosuppressive microenvironment that characterized liver metastases, thereby shedding light on the observed divergence.

SCLC, with its high tumor mutational burden, is notably responsive to immunotherapy [17, 18]. Multiple trials have substantiated the survival benefits of blocking the cytotoxic T-lymphocyte-associated protein 4 (CTLA-4), programmed death 1 (PD-1), and programmed death ligand 1 (PD-L1) axis, either in conjunction with chemotherapy or as maintenance therapy, for ES-SCLC patients [19–21]. However, several studies indicated that immunotherapy did not improve OS and PFS of ES-SCLC patients with liver metastases [22–24]. Consequently, disparities in the clinical advantages of immunotherapy in ES-SCLC patients with liver metastases have prompted thorough investigation. Our study contributed to the comprehension of this incongruity, demonstrating that first-line immunotherapy did not confer significant improvements in terms of PFS and OS to ES-SCLC patients with liver metastases. However, the observed P values of 0.05 suggested a potential trend towards improvement, warranting cautious interpretation and emphasizing the need for prospective studies.

The explanation for these findings may lie in the immunosuppressive microenvironment within liver metastases, which undermined the efficacy of immunotherapy [25]. Liver non-parenchymal cells present antigens to T-cells in a tolerogenic manner [26], leading to the apoptosis of activated antigen-specific T-cells upon interaction with monocyte-derived macrophages [27]. This phenomenon may account for the observed absence of immunotherapy benefits. Support for this explanation was found in a pooled analysis of non-small cell lung cancer patients with liver metastases, which revealed no discernible immunotherapy advantage (HR = 0.80, 95% CI: 0.49-1.31) [28].

A limitation of this study lies in its inability to differentiate patients with isolated liver metastases from those with metastases in other organs. This limitation underscored the necessity for judicious interpretation of the conclusions drawn. Additional research is indispensable to validate the findings presented in this systematic review and meta-analysis.

In conclusion, first-line immunotherapy plus chemotherapy significantly improved PFS and OS in ES-SCLC patients without liver metastases compared to chemotherapy alone. However, patients with liver metastases did not experience comparable benefits.
